# Morphine Enhances Doxorubicin-Induced Cardiotoxicity in the Rat

**DOI:** 10.1007/s12012-014-9249-z

**Published:** 2014-02-15

**Authors:** Lisa Drange Hole, Terje Hjalmar Larsen, Kjell Ove Fossan, Fredrik Limé, Jan Schjøtt

**Affiliations:** 1 Faculty of Medicine and Dentistry, Institute of Clinical Science, University of Bergen, 5021 Bergen, Norway; 2 Institute of Biomedicine, University of Bergen, 5021 Bergen, Norway; 3 Department of Heart Disease, Haukeland University Hospital, 5021 Bergen, Norway; 4 Laboratory of Clinical Biochemistry, Section of Clinical Pharmacology, Haukeland University Hospital, 5021 Bergen, Norway

**Keywords:** Doxorubicin, High-sensitivity cardiac troponin T, Hydrogen peroxide, Heart, Doxorubicinol, Cardiotoxicity

## Abstract

Interventions to reduce the cardiotoxicity of doxorubicin are clinically relevant. Pharmacological preconditioning mimicking ischemic preconditioning has been demonstrated with morphine and represents an acceptable clinical intervention. The purpose of this study was to examine if pretreatment in vivo with morphine could reduce doxorubicin-induced cardiotoxicity ex vivo in a rat model. Wistar rats were divided into six groups and pretreated with an intraperitoneal (i.p.) injection of 3 or 10 mg/kg morphine, 1 mg/kg naloxone and saline, 1 mg/kg naloxone and 3 mg/kg morphine or saline, 60 min before excision of the heart. Biochemical indices such as troponin T (TnT) and hydrogen peroxide (H_2_O_2_) in effluate were measured together with physiological parameters in Langendorff hearts before and after doxorubicin infusion (2 mg/mL 0.05 mL/min for 45 min). Myocardial content of doxorubicin was measured at the end of infusion. Pretreatment with morphine, irrespective of dosage, produced a significant loss in left ventricular-developed pressure and an increase of TnT and H_2_O_2_ in effluate before doxorubicin infusion (*p* < 0.05). Morphine also produced a significant increase in left ventricular end-diastolic pressure and an increase of TnT and H_2_O_2_ in effluate (*p* < 0.05) at the end of doxorubicin infusion. Naloxone, a non-selective opioid receptor antagonist, abolished the effects of morphine both before and after doxorubicin infusion. Morphine, irrespective of dosage, increased myocardial content of doxorubicin compared to pretreatment with saline (*p* < 0.05). Pretreatment with morphine is associated with a cardiodepressive effect and enhances cardiotoxicity of doxorubicin measured by increased myocardial accumulation of doxorubicin and physiological and biochemical indices. The negative effects observed in our rat model are abolished by naloxone.

## Introduction

The anthracycline doxorubicin is a frequently prescribed anticancer drug because of its effect on solid tumors as well as haematological malignancies. However, its pronounced cardiotoxicity limits long-term use and prevents effective anticancer therapy [1]. Dose reduction protocols to avoid the risk of delayed cardiotoxicity might be at the expense of the anticancer effect [2]. Generation of reactive oxygen species (ROS) and impaired calcium handling have been proposed as toxic mechanisms to explain both acute and delayed cardiotoxicity of anthracyclines [3–5].

Measurement of cardio-specific biomarkers can be a valid diagnostic tool for early identification, assessment and monitoring of cardiotoxicity [6]. Cardiac troponins have been suggested as valuable biomarkers of anthracycline cardiotoxicity, both in animal and clinical studies [7, 8], and they are advantageous because samples are obtained by a minimally invasive procedure and easily analyzed. However, few reported studies have characterized the cardiac troponin response in chemically induced chronic progressive myocardial injury such as we see with doxorubicin exposure [9]. The sensitivity and precision of the troponin T (TnT) assays have been improved in recent years and clinically approved high-sensitivity assays now detect cardiac troponins in nanogram per liter concentration.

Morphine is a non-selective opioid receptor agonist. Direct stimulation of myocardial δ_1_-opioid receptors leads to activation of mitochondrial K_ATP_-channels and a resultant increase in intracellular ROS in vitro. This is an important component of the signaling pathways by which morphine mimics preconditioning in cardiomyocytes [10]. Pharmacological preconditioning with morphine could represent an acceptable clinical intervention to reduce the cardiotoxicity of doxorubicin. Thus, it is of interest to study how morphine interacts with the toxic mechanisms of anthracyclines by measuring generation of ROS. To our knowledge, only one study has previously investigated the cardiac effect of intraperitoneal (i.p.) morphine before doxorubicin i.p. in rats, and this study found that morphine protects against doxorubicin cardiotoxicity [11]. A previous pilot study of 2-week duration, where we pretreated rats in vivo with i.p. morphine or saline 60 min before i.p. doxorubicin, every other day for 11 days, found that morphine, on the contrary, increased mortality in combination with doxorubicin [28].

In order to explore our negative findings in the pilot study, we pretreated rats in vivo with morphine before the hearts were isolated and exposed to doxorubicin ex vivo in a Langendorff model. Naloxone, a non-selective opioid receptor antagonist, was administered to investigate whether the potential effect of morphine would be abolished, or if naloxone itself had an effect. The aim of this study was to investigate the interaction of morphine and doxorubicin exclusively in the heart. Measurement of relevant biochemical indices such as TnT and hydrogen peroxide (H_2_O_2_) in effluate combined with physiological parameters and myocardial content of doxorubicin were included in the model.

## Materials and Methods

### Materials

Doxorubicin was purchased from Meda AS (Slemmestad, Norway), morphine, naloxone and pentobarbital from Haukeland Hospital Pharmacy (Bergen, Norway), heparin from Leo Pharma A/S (Oslo, Norway), and ingredients for the Krebs-Henseleit bicarbonate buffer from Merck KGaA (Darmstadt, Germany). This study conforms to the guide for the care and use of laboratory animals published by the US National Institutes of Health (NIH publication no. 85-23, revised 1996) and was approved by the Animal Care and User Committee in Norway.

### Animals

Male Wistar rats weighing 200 ± 20 g were purchased from Taconic (Ejby, Denmark). The animals were housed in grid-bottom metal wire cages in a room maintained at a 12 h light–dark cycle at a temperature of 20–22 °C. They were acclimatized for 2 weeks, housed four per cage and allowed free access to food pellets (Pellets rodent, Special Diets Services, Essex, UK) and tap water until injection of morphine, naloxone, saline or doxorubicin. The animals were separated in individual cages based on their respective treatment protocols.

### Langendorff Perfusion Model

The perfusion medium was a modified, oxygenated (95 % O_2_ and 5 % CO_2_) Krebs-Henseleit bicarbonate buffer (KHBB) (pH 7.4) containing in mM: 118.5 NaCl, 25.0 NaHCO_3_, 1.2 MgSO_4_, 4.7 KCl, 1.2 KH_2_PO_4_, 11.0 d-glucose, and 1.25 CaCl_2_. Hearts were excised after anesthesia of the rats with an i.p. injection of pentobarbital 50 mg/kg (0.1 mL/100 g bodyweight) and heparinized i.p. (0.1 mL 500 IU/100 g bodyweight). Anesthesia was evaluated by the pedal-withdrawal reflex. The heart was rapidly excised and immediately placed in cold (4 °C) KHBB to temporarily stop its beating and preserve it from ischemic injury prior to perfusion. The heart was mounted on a steel cannula placed in the aorta and perfused retrogradely in a Langendorff system with the use of thermostated (37 °C) reservoirs (Lauda, Lauda-Königshofen, Germany), perfusion lines and heart chamber. Volume-regulated flow (12.5 mL/min) was performed by use of an Alitea peristaltic pump (Alitea, Stockholm, Sweden). A perfusion pump (B. Braun, Melsungen, Germany) connected to a side arm of the aortic cannula was used for administration of doxorubicin or KHBB ex vivo. A water-filled latex balloon was placed in the left ventricle and connected to a pressure transducer (Memscap AS, Skoppum, Norway) for the recording of left ventricular pressure (LVDP) and secondarily derived contractility indices. Left ventricular end-diastolic pressure (LVEDP) was adjusted between 4 and 8 mmHg. A second pressure transducer was connected to a side arm on the aortic cannula for the recording of aortic pressure (AoP), as an index of coronary vascular resistance during volume-regulated perfusion. Pressure signals were amplified (Quadbridge, AD Instruments, London, UK) and recorded using a PowerLab data acquisition system (AD Instruments, East Sussex, UK). AoP, LVDP, LVEDP, left ventricular pressure first derivatives maximum (d*p*/d*t*_max_) and minimum (d*p*/d*t*_min_) were displayed and recorded. Pacing (300 beats per minute by electric stimulation of 5 V amplitude of 3 ms duration) during the doxorubicin or KHBB ex vivo protocol was obtained by placing one electrode on the right auricle and one on the steel cannula. Pacing was used to maintain a standard contractile response to the experimental drugs in the model not influenced by changes in heart rate and/or periods of arrhythmia. Effluate of KHBB mixed with doxorubicin was sampled for measurements of doxorubicin concentration in the perfusion system before mounting of the hearts. Effluate samples of 1 mL were collected in 1.5 mL polypropylene Eppendorf micro-test tubes (Eppendorf Vertrieb, Wesseling-Berzdorf, Germany) from each heart, at baseline and at the end of the perfusion protocol, and stored at 0º C, until analysis for TnT within 4 days of termination of the Langendorff protocol. Effluate samples of 1 mL were collected in Eppendorf tubes from each heart, at baseline and at the end of the perfusion protocol, and immediately analyzed for H_2_O_2_. Samples were placed in a thermostated (37 °C) Eppendorf rack heated by a Lauda reservoir (Lauda, Königshofen, Germany). At the end of the perfusion protocol, hearts were removed from the Langendorff system, and myocardial tissue from the left ventricle was dissected free and immediately frozen in liquid helium and stored at −80 °C until analysis of doxorubicin and doxorubicinol within 14 days of termination of the Langendorff protocol. All experiments and analysis were carried out between 7 a.m. and 7 p.m.

### Experimental Design

**Fig. 1:**
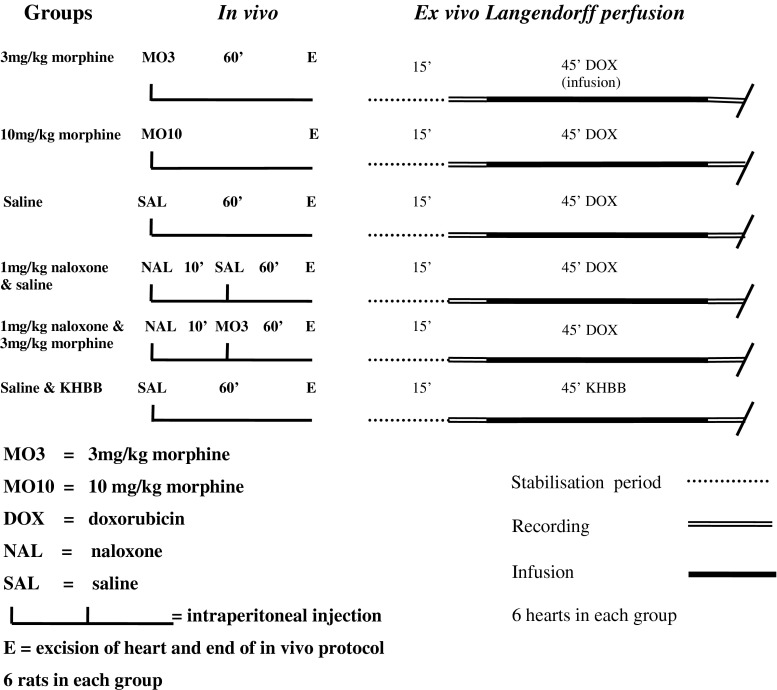
Treatment and perfusion protocols

Rats were randomly divided into three morphine treatment groups (3 or 10 mg/kg morphine or 3 mg/kg morphine and 1 mg/kg naloxone) and one naloxone group (1 mg/kg). Naloxone was administrated 10 min before morphine or saline. Two control groups were included: One pretreated with 0.9 % saline before doxorubicin infusion, a control for pretreatment, and one pretreated with 0.9 % saline before KHBB infusion, a control for the experimental model. All pretreatment was administrated by i.p. injection. Each group included six rats. 60 min after pretreatment, hearts were excised and Langendorff perfused with the following protocol: 15-min stabilization period, after which physiological parameters were recorded for 5 min and effluate was collected. Hearts then received 45 min of infusion with undiluted 2 mg/mL doxorubicin at a rate of 0.05 mL/min (or KHBB for the control group). Physiological data were collected immediately after the infusion was terminated. Ultimately, a 5-min wash-out period followed after which we collected effluates, and the Langendorff protocol was terminated. Treatment protocols and perfusion protocols are illustrated in Fig. 1.

### Quantification of Doxorubicin and Doxorubicinol

Doxorubicin and doxorubicinol were quantified by high-performance liquid chromatography (HPLC-MS/MS) (1200 series RRLC, Agilent Technologies, USA) coupled with an Agilent 6410 triple quadrupole mass spectrometer using positive electrospray ionization (Agilent Technologies, USA). Frozen left ventricular tissue was minced and weighted out in a glass tube with a screw cap and homogenized in physiological saline (2 mL/100 mg tissue) with a tissue homogenizer (Ultra Turrax, Sigma Aldrich, Germany). 1000 μL of sample was added to 100 μL of daunorubicin as internal standard (IS) and 200 μL of buffer (1M TRIZMA, pH 11.1) and mixed well before extraction with 4 mL ethyl acetate/heptane (80/20 vol/vol). The samples were mixed using a rotary blender for 15 min and then centrifuged at 3500 rpm for 10 min at 10 °C. The organic phase was evaporated to dryness at 50 °C under nitrogen then dissolved in 100 μL of methanol followed by 100 μL of distilled water. The extract was mixed thoroughly and transferred to silanized vials before analysis. 25 μL of extract was injected and separated on a Zorbax SB-Aq (2.1 × 50 mm, 1.8 μm particles, Agilent Technologies, USA) column using gradient elution with acetonitrile and 0.1 % formic acid in water. Quantification were performed using multiple reaction monitoring mode at *m*/*z* 546.1 → 363.1 for doxorubicinol, *m*/*z* 544.1 → 361.1 for doxorubicin and *m*/*z* 528.1 → 321.1 for daunorubicin (IS).

### Effluate Content of H_2_O_2_

H_2_O_2_ in cardiac effluate was measured using an Apollo 4000 electrochemical detection system (World Precision Instruments, Sarasota, FL, USA). The electrode was calibrated using nine serial dilutions of H_2_O_2_ in phosphate-buffered saline with added aniline. The current recorded from the effluate was then calculated as μM H_2_O_2_. Samples were kept at 37 °C during measurement. The electrode was allowed 3 min of stabilization and 1 min of recording.

### Effluate Content of TnT

TnT in cardiac effluate was measured using an Elecsys 2010 immunoassay analyzer (Roche Diagnostics Norway AS, Oslo, Norway), based on the sandwich principle. Total duration of assay: 9 min. First incubation: 50 μL of sample, a biotinylated monoclonal cardiac TnT-specific antibody, and a monoclonal cardiac TnT-specific antibody labeled with a ruthenium complex (tris(2,2-bipyridyl)ruthenium(II)-complex(Ru(bpy))) reacted to form a sandwich complex. Second incubation: After addition of streptavidin-coated microparticles, the complex became bound to the solid phase via interaction of biotin and streptavidin. The reaction mixture was aspirated into the measuring cell where the microparticles were magnetically captured onto the surface of the electrode. Unbound substances were then removed with ProCell. Application of a voltage to the electrode then induced chemiluminescent emission which was measured by a photomultiplier. Results were determined via a calibration curve which was instrument specifically generated by 2-point calibration and a master curve (5-point calibration) provided via the reagent barcode. Detection limit was 5.0 ng/L.

### Statistics

All results are reported as mean values ± standard deviation (SD) in tables. Data were collected at two time points: before doxorubicin infusion and at the end of infusion. Groups were compared with regards to parameters with a one-way analysis of variance and subsequently fisher’s protected least-significant difference test. SPSS for windows version 17.0 was used, and *p* < 0.05 was considered statistically significant.

## Results

All physiological results are presented in Table 1 and pharmacological and biochemical results in Table 2. Results from comparison of the two morphine groups have been left out of Tables 1 and 2 as they showed no statistically significant difference between the two different morphine doses.

**Table 1:** Physiological results

	Group 1 3 mg/kg morphine	Group 2 10 mg/kg morphine	Group 3 Saline	Group 4 1 mg/kg naloxone and saline	Group 5 1 mg/kg naloxone and 3 mg/kg morphine	Group 6 Saline control
LVDP baseline (mmHg)	84.3 ± 8.3*	76.8 ± 5.8*	149.8 ± 10.7	145.3 ± 7.7	139.8 ± 9.1	145.7 ± 9.6
LVDP end (mmHg)	52.8 ± 11.1*	50.7 ± 10.9*	102.5 ± 5.9	99.8 ± 7.7	100.3 ± 11.1	135.5 ± 6.6*
LVDP (%)	62.9 ± 13.3	66.2 ± 14.7	68.8 ± 7.5	68.6 ± 2.6	71.9 ± 7.9	93.1 ± 2.4*
LVEDP baseline (mmHg)	5.6 ± 1.6	6.1 ± 1.3	6.2 ± 1.5	5.6 ± 1.4	6.5 ± 1.3	7.4 ± 1.0
LVEDP end (mmHg)	23.2 ± 1.8*	22.4 ± 1.8*	13.2 ± 1.7	13.5 ± 1.8	12.9 ± 1.4	8.6 ± 0.6*
LVEDP (%)	440.7 ± 124.9*	381.2 ± 94.1*	219.2 ± 44.2	254.4 ± 70.7	206.1 ± 53.9	117.7 ± 14.5
AoP baseline (mmHg)	92.5 ± 4.4*	89.5 ± 6.1*	103.3 ± 4.1	107.0 ± 3.1	104.2 ± 5.2	107.7 ± 2.7
AoP end (mmHg)	150.3 ± 5.8*	151.5 ± 6.6*	164.0 ± 5.8	159.2 ± 7.0	160.0 ± 7.2	114.6 ± 4.6*
AoP (%)	162.9 ± 11.9	169.7 ± 10.1	158.8 ± 6.5	148.7 ± 2.9	154.1 ± 12.6	106.4 ± 2.9*
d*p*/d*t*_max_ baseline (mmHg)	2,855.3 ± 378.5*	2,733.5 ± 458.5*	4,677.7 ± 306.2	4,934.8 ± 205.7	4,710.2 ± 125.5	4,788.7 ± 291.4
d*p*/d*t*_max_ end (mmHg)	1,551.0 ± 293.8*	1,542.3 ± 278.9*	2,873.8 ± 418.4	2,902.8 ± 423.8	2,779.5 ± 588.1	4,378.3 ± 449.5*
d*p*/d*t*_max_ (%)	54.7 ± 10.2	58.0 ± 15.2	61.8 ± 10.6	58.7 ± 7.4	58.9 ± 12.2	91.3 ± 4.6*
d*p*/d*t*_min_ baseline (mmHg)	−1,767.8 ± 125.9*	−1,853.0 ± 164.8*	−3,191.5 ± 279.3	−3,184.8 ± 308.9	−3,049.5 ± 263.5	−3,746.5 ± 220.2*
d*p*/d*t*_min_ end (mmHg)	−1,170.7 ± 107.9*	−1,326.2 ± 143.2*	−2,148.5 ± 378.8	−2,186.8 ± 196.6	−1,970.5 ± 177.9	−3,587.8 ± 282.0*
d*p*/d*t*_min_ (%)	66.3 ± 5.4	71.9 ± 8.5	67.1 ± 8.5	69.5 ± 11.6	65.1 ± 9.0	95.8 ± 4.5*

Values presented as mean ± SD

* Significantly different from hearts in group 3, *p* < 0.05

**Table 2:** Pharmacological and biochemical results

	Group 1 3 mg/kg morphine	Group 2 10 mg/kg morphine	Group 3 Saline	Group 4 1 mg/kg naloxone and Saline	Group 5 1 mg/kg naloxone and 3 mg/kg morphine	Group 6 Saline control
Doxorubicin myocardial tissue concentration at the end of experiments (nmol/g)	205.6 ± 27.6*	213.7 ± 11.1*	168.5 ± 9.5	185.2 ± 7.7	188.8 ± 11.7	0.0
Doxorubicinol myocardial tissue concentration at the end of experiments (nmol/g)	3.1 ± 0.2	4.0 ± 1.4	3.2 ± 0.5	1.4 ± 0.5*	2.1 ± 0.5*	0.0
Troponin T in effluate before doxorubicin exposure (ng/L)	63.3 ± 20.7*	84.8 ± 20.3*	22.7 ± 7.4	39.0 ± 15.7	47.0 ± 4.6	16.8 ± 3.8
Troponin T in effluate after doxorubicin exposure (ng/L)	321.5 ± 108.5*	473.5 ± 117.6*	129.2 ± 6.6	177.8 ± 4.6	187.2 ± 8.2	36.0 ± 10.0
H_2_O_2_ in effluate before doxorubicin exposure (μM)	33.6 ± 0.5*	39.5 ± 5.8*	28.4 ± 2.1	26.9 ± 3.6	26.9 ± 3.6	27.6 ± 3.3
H_2_O_2_ in effluate after doxorubicin exposure (μM)	40.9 ± 3.6*	45.6 ± 1.3*	34.9 ± 1.2	33.3 ± 1.8	33.9 ± 2.5	28.8 ± 3.4*

Values presented as mean ± SD

* Significantly different from hearts in group 3, *p* < 0.0.5

Reduction in cardiac function measured by LVDP and release of troponin and H_2_O_2_ were evident in hearts from morphine-treated rats before infusion with doxorubicin was started. Hearts from rats pretreated with 3 mg/kg (84.3 ± 8.3 mmHg) and 10 mg/kg (76.8 ± 5.8 mmHg) morphine demonstrated significantly (*p* < 0.05) lower values of LVDP at baseline, compared with hearts pretreated with saline (149.8 ± 10.7 mmHg). There was no difference in LVDP at baseline in hearts from rats pretreated with naloxone, or naloxone and 3 mg/kg morphine compared with hearts pretreated with saline.

The reduction in cardiac function measured by LVDP and release of troponin and H_2_O_2_ were more pronounced in hearts from morphine-treated rats after doxorubicin exposure, compared with hearts from saline or naloxone-treated rats. Hearts from rats pretreated with 3 mg/kg (52.8 ± 11.1 mmHg) and 10 mg/kg (50.7 ± 10.9 mmHg) morphine demonstrated significantly (*p* < 0.05) lower values of LVDP at the end of the doxorubicin infusion, compared with hearts pretreated with saline (102.5 ± 5.9 mmHg). There was no difference in LVDP at the end of the doxorubicin infusion in hearts from rats pretreated with naloxone, or naloxone and 3 mg/kg morphine, compared with hearts pretreated with saline. However, reduction in physiological parameters in per cent was similar in all groups 1–5, irrespective of pretreatment during 45 min of doxorubicin infusion.

At the end of infusion, hearts from rats pretreated with 3 mg/kg (23.2 ± 1.8 mmHg) and 10 mg/kg (22.4 ± 1.8 mmHg) morphine demonstrated significantly (*p* < 0.05) higher LVEDP, compared with hearts pretreated with saline (13.2 ± 1.7 mmHg). There was no significant difference in LVEDP in the naloxone-pretreated groups, compared with the saline-pretreated group, at the end of infusion.

Hearts from rats pretreated with 3 mg/kg (33.6 ± 0.5 μM) and 10 mg/kg (39.5 ± 5.8 μM) morphine demonstrated significantly (*p* < 0.05) increased release of H_2_O_2_ in effluate compared with hearts pretreated with saline (28.4 ± 2.1 μM), at baseline. Similarly, H_2_O_2_ in effluate from rats pretreated with 3 mg/kg (40.9 ± 3.6 μM) and 10 mg/kg (45.6 ± 1.3 μM) morphine was increased at the end of infusion, compared with H_2_O_2_ in effluate from rats pretreated with saline (34.9 ± 1.2 μM).

Hearts from rats pretreated with 3 mg/kg (63.3 ± 20.7 ng/L) and 10 mg/kg (84.8 ± 20.3 ng/L) morphine demonstrated significantly (*p* < 0.05) higher values of TnT at baseline, compared with hearts pretreated with saline (22.7 ± 7.4 ng/L). There was no difference in TnT at baseline in hearts from rats pretreated with naloxone, or naloxone and 3 mg/kg morphine compared with hearts pretreated with saline. Similarly, hearts from rats pretreated with 3 mg/kg (321.5 ± 108.5 ng/L) and 10 mg/kg (473.5 ± 117.6 ng/L) morphine demonstrated significantly (*p* < 0.05) higher values of TnT at the end of the doxorubicin infusion, compared with hearts pretreated with saline (129.2 ± 6.6 ng/L). There was no difference in TnT at the end of the doxorubicin infusion in hearts from rats pretreated with naloxone, or naloxone and 3 mg/kg morphine compared with hearts pretreated with saline.

Myocardial content of doxorubicin was significantly (*p* < 0.05) higher in the 3 mg/kg (205.6 ± 27.6 nmol/g) and 10 mg/kg (213.7 ± 11.1 nmol/g) morphine group compared with the saline-pretreated group (168.5 ± 9.5 nmol/g), at the end of infusion. The concentration of doxorubicin in the perfusate was measured to a mean of 5.42 ± 1.98 μM based on samples (*n* = 30) from the aortic cannula of the Langendorff system without hearts mounted. Hearts were paced to 300 beats per minute; thus, data on heart rate have been left out of Table 1.

## Discussion and Conclusion

The main observation in the present results is that the increased mortality associated with morphine could be related to enhanced cardiotoxicity. In the pilot study [28], we established an in vivo model with three groups: eight rats were pretreated with an i.p. injection of 3 mg/kg morphine, and 8 rats were pretreated with an i.p. injection of 0.9 % saline, 60 min prior to a 3 mg/kg doxorubicin i.p. injection every other day for 11 days, up to a cumulative dose of 12 mg/kg doxorubicin. Five rats were pretreated with an i.p. injection of 0.9 % saline 60 min prior to an i.p. injection of 0.9 % saline every other day for 11 days. On day 12, hearts from the three groups were planned to be excised and Langendorff perfused for comparison of physiological and biochemical indices of cardiotoxicity. However, 6 out of 8 rats in the group pretreated with morphine died before day 12 and did not complete the treatment protocol. Mortality was evident in the group by day 9 (*n* = 1) and by days 10 and 11 (*n* = 5). The remaining two rats were moribund and euthanasia was performed. Thus, physiological parameters from rats pretreated with morphine were not available for comparison with the other groups, and myocardial tissue was discarded due to risk of postmortem redistribution of drugs. Based on these results, we hypothesized that the interaction between doxorubicin and morphine could result in increased cardiotoxicity. The results from the pilot study were unexpected because a similar study [11] found morphine to be protective against doxorubicin-induced cardiotoxicity in rat. This study pretreated rats with an i.p. dose of 10 mg/kg morphine 30 min prior to doxorubicin. Doxorubicin (1.25 mg/kg i.p.) was administrated four times per week for 4 weeks, with a total cumulative dose of 20 mg/kg. Cardioprotective efficacy of morphine was performed by analyzing the electrocardiographic parameters (QRS complexes and ST segments) and contractility force of left ventricular papillary muscle, and these parameters were improved in rats pretreated with morphine. Morphine also reduced mortality in this study. We measured LVDP and derived indices. The morphine dose is the same in our study. However, we delivered it 60 min prior to doxorubicin. In early preconditioning, a memory phase of up to 2 h after trigging has been reported in which protection is demonstrated [12, 13]. Theoretically, this should allow for opioid receptor activation in the memory phase of preconditioning in the rat myocardium.

Enhanced mortality by a combination of morphine and doxorubicin has previously been demonstrated in mice where morphine pretreatment caused a dose-dependent increase in plasma doxorubicin [14]. In our study, rats were pretreated in vivo with morphine before the hearts were isolated and exposed to doxorubicin ex vivo. Pretreatment with morphine in vivo, irrespective of dose, is associated with a cardiodepressive effect in isolated hearts combined with increased release of H_2_O_2_ and TnT. After exposure to doxorubicin ex vivo, isolated hearts from rats pretreated with morphine, irrespective of dose, demonstrate increased release of H_2_O_2_ and TnT, increased myocardial contracture and increased myocardial accumulation of doxorubicin. These effects were abolished when naloxone was administrated before morphine in pretreatment. The enhanced cardiotoxicity of pretreatment with morphine was in particular evident by LVEDP. Myocardial contracture assessed by an increase in LVEDP suggests that pretreatment with morphine enhanced diastolic dysfunction during doxorubicin infusion. Diastolic dysfunction is proposed to reflect impaired calcium handling which together with generation of oxygen species are proposed as toxic mechanisms of anthracyclines [3]. Interestingly, diastolic dysfunction is proposed to precede systolic dysfunction in chronic anthracycline cardiotoxicity and suggested as an early marker of subsequent heart failure in the clinic [3].

By using i.p. administration of 4 mg/kg morphine, a study on rats found peak plasma concentrations of about 1 μM after 8 min with a half-life of about 46 min [15]. Theoretically, this should allow for opioid receptor activation in the memory phase of preconditioning in the rat myocardium. In early preconditioning, a memory phase of up to 2 h after trigging has been reported in which protection is demonstrated [12, 13]. We used a dose of 3 mg/kg i.p and added a dosage of morphine of 10 mg/kg as reported by others [11, 16, 17], to investigate whether the dose used in the pilot study was too low to illicit a protective effect. However, both 3 and 10 mg/kg morphine i.p. had a cardiodepressive effect prior to exposure to doxorubicin. In this present study, cardiodepression with a theoretical morphine peak plasma concentration of approximately 1 μM is in accordance with previous observations [18].

In vitro studies have shown that opioids directly decrease the contractile response of isolated ventricular cardiomyocytes to electrical stimulation [19]. One hypothesis explaining morphine-induced decreased contractility is that morphine induced the increase in generation of free radicals that we measured at baseline. A study of pretreatment of chick embryonic ventricular myocytes with 1 μM morphine before 1 h of ischemia and 3 h of reoxygenation found a two-fold increased free radical production before ischemia compared with controls [20]. The increase in free radical signals with morphine was abolished by 5-hydroxydecanoate, a selective mitochondrial K_ATP_-channel antagonist [10]. We observed that the cardiodepressive effect was associated with increased release of H_2_O_2_ in morphine-treated hearts. However, after doxorubicin infusion, this was associated with further increased release of hydrogen peroxide and increased myocardial contracture evident by increased LVEDP. This raises the question whether an increase in generation of free radicals induced by morphine not only induced a cardiodepressive effect prior to doxorubicin, but produced additive myocardial damage to that produced by doxorubicin. H_2_O_2_ is an important by-product of oxidative metabolism and is a major contributor in oxidative stress-induced functional and metabolic dysfunction [21, 22]. An experiment that exposed isolated perfused rat hearts to 200 μM H_2_O_2_ for 30 min resulted in a time-dependent depression of myocardial contractility and a 1000 % elevation in LVEDP [4]. Similar results have been reported in isolated rat hearts [23, 24]. We found a depression in LVDP of approximately 40 % in the morphine groups and 30 % in the saline-pretreated group after 45 min of doxorubicin exposure. This effect was associated with approximately 400 % elevation in LVEDP in the morphine groups, irrespective of dose, compared with 200 % in the saline group with levels of H_2_O_2_ in the range of 35–45 μM. Thus, our results suggest that additive free radical damage generated by morphine and doxorubicin could result in contractile dysfunction. H_2_O_2_ mediates increased endothelial permeability and may increase the extravasation of doxorubicin and alter its distribution in the myocardium [25, 26]. Thus, a potential change in endothelial permeability due to increased H_2_O_2_ can explain the increased content of doxorubicin in hearts from rats pretreated with morphine in our study.

The concentration of doxorubicin in the perfusate was measured to 5.42 ± 1.98 μM. A non-physiological concentration could mask a protective effect of morphine. However, C_max_ of plasma doxorubicin following a 30 mg/m^2^ i.v. bolus dose in humans is reported to be 3 μmol/L, with cellular levels 30–100-fold higher than that of the plasma [27]. We do not know in which compartment of the myocardium the accumulation of doxorubicin took place. The main coronary vascular bed is unlikely due to our wash-out procedure. Furthermore, the reduced contractile function is not due to vasoconstrictive effects since we used a model with volume-regulated perfusion. However, doxorubicin could accumulate in the extracellular space as well as in the cardiomyocytes. The reduced contractile function suggests that at least a part of the accumulation affects the cardiomyocytes. We observed that H_2_O_2_ levels are altered by morphine in combination with doxorubicin. However, there is insufficient assessment of why this might lead to enhanced damage, and of the possibility that other reactive species may also be involved in the effects seen. Further studies are needed to assess this.

The Langendorff model bypasses the pharmacokinetic phase associated with drug metabolism and only measures the direct action of the drug in the myocardium. The interaction of morphine and doxorubicin in vivo could involve systemic effects that secondarily influenced the function of the heart. In the present study, we wanted to investigate how doxorubicin affects a heart that has already been exposed to morphine in vivo based on our findings in the pilot study where morphine was administrated in a similar way. Ideally, heart functional parameters could be measured in vivo after morphine and subsequent doxorubicin administration, but direct cardiotoxic effects could be difficult to separate from indirect systemic effects. Furthermore, both morphine and doxorubicin could be administrated in sequence in Langendorff hearts to study their effects exclusively in the heart, but pharmacokinetic effects would lack as mentioned above.

In conclusion, we found that pretreatment with morphine is associated with a cardiodepressive effect in isolated rat hearts combined with increased release of H_2_O_2_ and TnT. After exposure to doxorubicin ex vivo, isolated hearts from rats pretreated with morphine are associated with increased release of H_2_O_2_ and TnT, increased myocardial contracture and accumulation of doxorubicin. Morphine increases intracellular free radical signals, and this is an important component of the signaling pathways by which morphine mimics preconditioning in cardiomyocytes. However, results in our pilot study and the present study suggest that these pathways, although promising interventions to reduce cardiotoxicity of anthracyclines, are also associated with risk of additional damage in the rats.
